# Clinical Characterization of Ulcerative Colitis in Patients with Primary Sclerosing Cholangitis

**DOI:** 10.1155/2020/7969628

**Published:** 2020-11-07

**Authors:** Shun Murasugi, Ayumi Ito, Teppei Omori, Shinichi Nakamura, Katsutoshi Tokushige

**Affiliations:** Department of Gastroenterology, Tokyo Women's Medical University, Tokyo 162-8666, Japan

## Abstract

**Objectives:**

The clinical/colonoscopic features of ulcerative colitis (UC) associated with primary sclerosing cholangitis (PSC), the prognostic impact of UC, and the utility of UC screening in PSC patients are unknown. We characterized UC associated with PSC and assessed UC's impact on the prognosis of PSC and the importance of colonoscopic UC screening in PSC patients.

**Methods:**

We retrospectively analyzed the cases of 77 patients treated for PSC at a single center (April 2000–July 2019). We reviewed the clinical/colonoscopic profiles of the concurrent UC patients and compared the clinical profiles, survival, and primary causes of death between the patients with/without UC (*n* = 35/*n* = 42). The details of all patients' colonoscopies were reviewed.

**Results:**

The concurrent UC group: 17 men, 18 women, diagnosed with PSC at the mean (SD) age of 36 (17) years; 21 patients (60%) had no UC symptoms. Colonoscopy revealed pancolitis in all patients, predominantly affecting the right-sided colon in 30 patients (86%). Lesions were scattered. Backwash ileitis (*n* = 13, 37%) and rectal sparing (*n* = 18, 51%) were observed. Most patients had mild UC; some had moderate or more severe UC (median Ulcerative Colitis Endoscopic Index of Severity (UCEIS) score 2; range, 1–5). Ludwig's stage determined by liver biopsy did not correlate with the Mayo endoscopic score for UC. The patients with UC were diagnosed with PSC at a significantly younger age than those without UC (mean (SD), 36 [17] years vs. 55 [19] years, *p* < 0.0001) and had a significantly higher 5-year survival rate (97.1% vs. 70.5%, *p* = 0.0028). UC was detected in 19 of 34 asymptomatic patients (56%) who underwent colonoscopy screening.

**Conclusions:**

Our cohort's clinical/colonoscopic features of UC associated with PSC are more moderate or severe UC than previous cases. The coexistence of UC might affect the prognosis of PSC. In this regard, colonoscopy in PSC patients is an important examination for determining prognosis. There is also asymptomatic UC in patients with PSC. In this regard, screening for colonoscopy in PSC patients is essential. When a diagnosis of PSC is made, immediate colonoscopy is a priority with UC complications in mind.

## 1. Introduction

Primary sclerosing cholangitis (PSC) is a chronic cholestatic liver disease characterized by diffuse multiple structuring of the intrahepatic and extrahepatic bile ducts [1]. Japan has an estimated 2,300 PSC patients, and the prevalence of PSC is 1.8 per 100,000 people, having almost doubled over the past 11 years [2]. Western countries have higher prevalence rates of PSC, and the rates are also reported to be increasing there [3]. PSC has a poor prognosis because of the high risk of recurrence after liver transplantation [4], which is the only radical treatment for this condition. There are no effective medical treatments for PSC.

**Figure 1:**
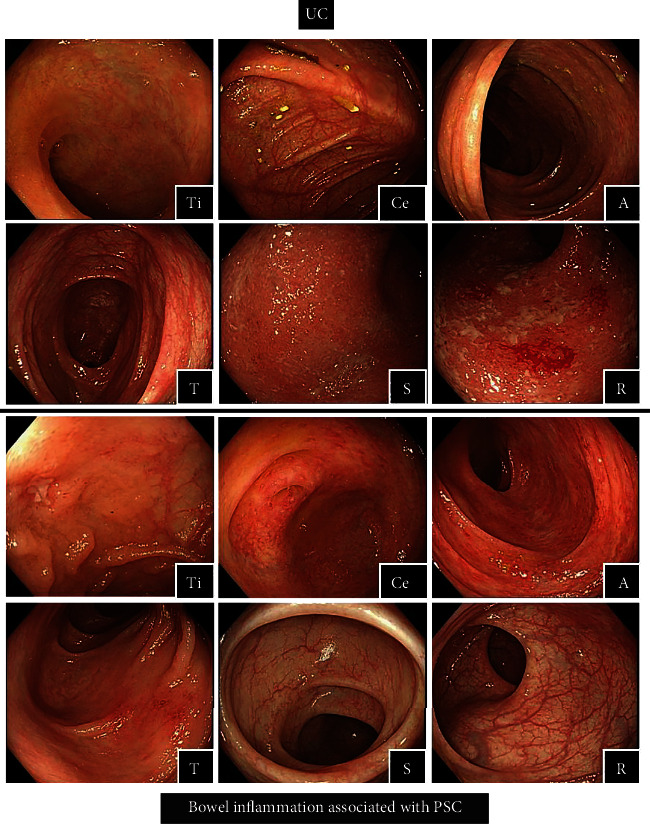
Colonoscopy of ulcerative colitis and bowel inflammation associated with PSC Ti: terminal ileum, Ce: cecum, A: ascending colon, T: transverse colon, S: sigmoid colon, and R: rectum.

PSC is closely related to inflammatory bowel disease (IBD) [1–5]. The most common type of IBD associated with PSC is ulcerative colitis (UC) [6–8]. Because UC is becoming more prevalent in Japan [9], IBD associated with PSC may also become more prevalent in Japan. It is thus likely that it will become important to diagnose and treat IBD in PSC patients in Japan. UC associated with PSC is known to have colonoscopic features that are distinct from those of classical UC without PSC (Table 1, Figure 1). In addition, UC associated with PSC is mostly mild and is occasionally followed up as nonspecific colitis. Clinicians should be aware of these and other differences between UC with and without underlying PSC.

The most common causes of death in PSC patients include liver failure due to the progression of PSC, infections, and malignancies [12]. Part of the significance of diagnosing and treating IBD associated with PSC comes from the fact that patients with IBD are at an increased risk of developing colorectal cancer (CRC). CRC has been identified as a significant prognostic factor for PSC [13], although it remains to be determined whether the coexistence of IBD has an impact on the prognosis of PSC [14, 15].

To more clearly determine the importance of diagnosing and treating IBD associated with PSC, we studied the clinical and colonoscopic features of UC associated with PSC, the impact of UC on the prognosis of underlying PSC, and the clinical significance of colonoscopic UC screening in PSC patients.

## 2. Materials and Methods

We retrospectively analyzed the cases of 77 patients treated for PSC at Tokyo Women's Medical University Hospital during the period from April 1, 2000 to July 31, 2019. The medical records of 35 patients with concurrent UC were reviewed to clarify the clinical features of UC associated with PSC (e.g., clinical course and colonoscopic findings). We collected data on the following parameters: sex, age at the diagnosis of PSC, age at the diagnosis of UC, the diagnostic sequence of UC in relation to PSC, the presence/absence of symptoms of UC, the extent of UC, the location of inflammation, the presence/absence of backwash ileitis [16], the presence/absence of rectal sparing [17], the Lichtiger Clinical Activity Index (CAI) [18], the endoscopic UC severity score (i.e., the Mayo endoscopic score for UC [19] and the Ulcerative Colitis Endoscopic Index of Severity [UCEIS] score [20]), Ludwig's stage determined by liver biopsy [21], and the treatments provided for UC and PSC.

The following characteristics of the patients with UC (*n* = 35) and those without UC (*n* = 42) were then compared: sex, age at the diagnosis of PSC, whether the patient underwent colonoscopy screening, the location of inflammation, the Child-Pugh classification [22, 23], Ludwig's stage determined by liver biopsy, treatments provided for PSC, biochemical parameters of hepatic function at diagnosis of PSC (i.e., the prothrombin time (PT) percentage activity, albumin, total bilirubin, aspartate aminotransferase (AST), alanine aminotransferase (ALT), and alkaline phosphatase (ALP)), serum creatinine, serum sodium, survival, primary cause of death, and the PSC follow-up period. The endpoint events for the calculation of the technical survival rate included liver transplantation and death. The PSC diagnosis followed the established guidelines [24].

In the entire population of PSC patients, we also investigated whether individual patients underwent a colonoscopy, the reasons for the colonoscopy, and whether the colonoscopy detected UC.

All data are expressed as the number of patients or the mean (SD) except for the clinical and endoscopic disease activity index scores, which are expressed as the median (range). Data were compared between the two groups by the chi-square test or Wilcoxon's test. Survival rates were estimated by the Kaplan-Meier method and compared between the two groups by the log-rank test. *p* values < 0.05 were considered significant. Statistical analyses were performed with JMP Pro14 Software (SAS Institute, Cary, NC). The study was approved by the Institutional Ethics Review Board (approval no. 5167).

## 3. Results

### 3.1. The Characteristics of UC Associated with PSC

We identified 35 patients with concurrent UC (17 men and 18 women). The mean (SD) age at the diagnosis of PSC was 36 (17) years. The diagnosis of UC was after (*n* = 10, 29%), before (*n* = 10, 29%), or concurrent with (*n* = 15, 42%) the diagnosis of PSC (Table 2). Twenty-one of the 35 patients with concurrent UC (60%) had no symptoms of UC. Colonoscopy revealed pancolitis in all patients, which predominantly affected the right-sided colon in 30 patients (86%). The UC was characterized by scattered lesions with backwash ileitis in 13 patients (37%) and rectal sparing in 18 patients (51%). The median Lichtiger CAI score was 4 (range 2–8). Endoscopically, most of the patients had mild UC, but some had moderate or more severe UC (i.e., a median Mayo score of 1 (range 1–3) and a median UCEIS score of 2 (range, 1–5)).

In the group of 23 patients who underwent a liver biopsy, Ludwig's stage determined by liver biopsy did not correlate with the endoscopic UC severity scores. The treatments provided included ursodeoxycholic acid (*n* = 31), which was coadministered with 5-aminosalicylic aid (5-ASA; *n* = 24, 69%), prednisolone (PSL; *n* = 12, 34%), cytapheresis (CAP; *n* = 5, 14%), or azathioprine (*n* = 3, 9%). Two patients (5.7%) developed CRC and underwent a total colectomy.

### 3.2. Comparison of the PSC Patients with and without UC

#### 3.2.1. Clinical Features

Our comparison of the clinical profiles of the 35 patients with UC with those of the 42 patients without UC revealed significant differences with respect to the mean (SD) age at the diagnosis of PSC (36 [17] years old in the UC group vs. 55 [19] years in the without-UC group; *p* < 0.0001) and the percentage of patients who underwent a colonoscopy (100% vs. 48%, respectively; *p* < 0.0001). As listed in Table 3, at the time that PSC was diagnosed, the patients with UC had numerically lower values than the patients without UC for the following biochemical parameters of hepatic function, although the differences were not significant: total bilirubin, AST, ALT, and ALP.

#### 3.2.2. Survival Rate and Causes of Death

**Figure 2:**
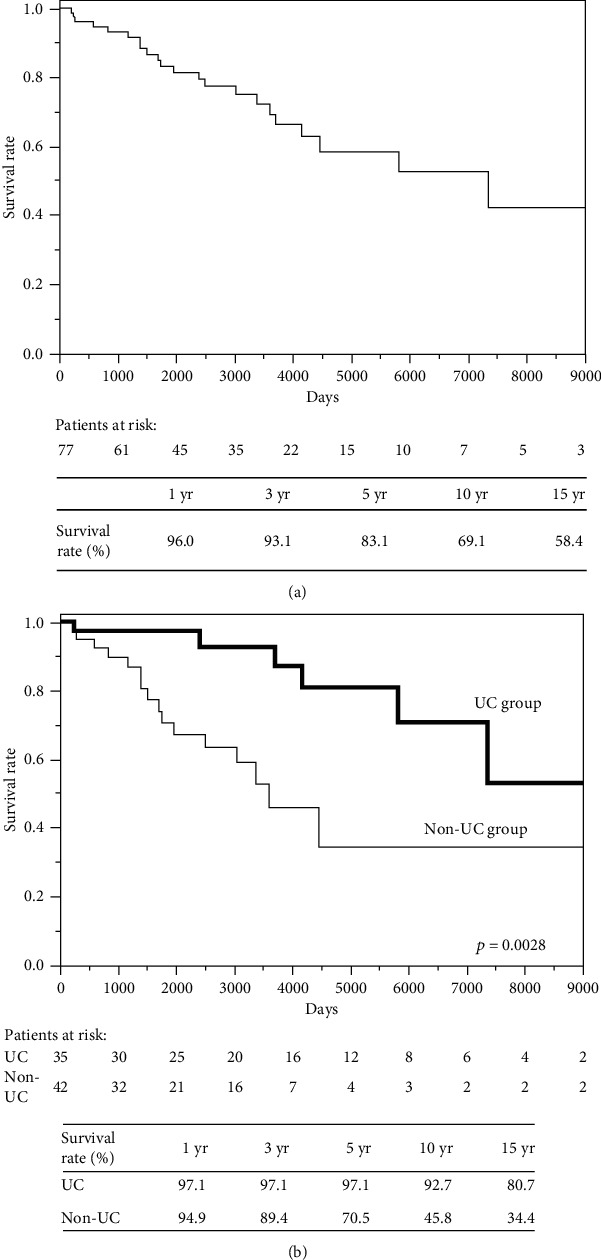
(a) Survival rate: the total PSC cohort (*n* = 77). (b) Survival rate: PSC and PSC complicated by UC.

The 10-year survival rate in the entire population of PSC patients was 69.1% (Figure 2(a)). The patients with UC had a significantly higher 5-year survival rate compared to the patients without UC (97.1% vs. 70.5%, respectively; *p* = 0.0038; Figure 2(b)). Thirteen patients died, including nine (69.2%) who died of liver failure, three (23.1%) from an infection, and one patient (7.7%) who died due to ruptured esophageal varices. No patients died of CRC.

As an additional study, the survival rate was examined in 20 patients excluding patients who did not undergo colonoscopy from non-UC, and similar results were obtained (Supplementary Figure S1).

#### 3.2.3. The Reasons to Undergo a Colonoscopy

Of the entire cohort of 77 PSC patients, 55 (71%) (35 with UC, 20 without UC) underwent a colonoscopy. Of the 22 patients who did not undergo a colonoscopy, a colonoscopy had been recommended to 16 (72.7%) but they refused, and it was prevented by the presence of ascites in five patients (22.7%) and by a poor general condition in one patient (4.5%). Among the 35 PSC patients with UC, a colonoscopy was prompted by any symptom of UC (*n* = 14, 40.0%) or by asymptomatic occult blood-positive stools (*n* = 2, 5.7%). In the remaining 19 patients (54.3%), a colonoscopy was performed only for screening purposes in the absence of symptoms of UC and occult blood-positive stools.

Among the 20 patients without UC, a colonoscopy was prompted by any symptom of UC (*n* = 2, 10%) or by asymptomatic occult blood-positive stools (*n* = 3, 15%). In the remaining 15 patients (75%), a colonoscopy was performed only for screening purposes in the absence of symptoms of UC and occult blood-positive stools. In total, 34 of the 55 patients who underwent a colonoscopy did so for screening purposes, and 19 of them (56%) had colonoscopic findings indicating a diagnosis of UC (Table 4).

## 4. Discussion

### 4.1. Features of UC Associated with PSC

Consistent with the PSC patients in previous studies (Table 1), our PSC patients with UC presented with colonoscopic features that were distinct from those of classical UC; i.e., they had pancolitis (which affected predominantly the right-sided colon) and scattered lesions, occasionally with backwash ileitis and rectal sparing. Several studies have suggested that PSC has unique dysbiosis characteristics, unlike UC [25–27]. These unique features may be the cause of the difference in characteristics. Compared to the previous studies, more of our PSC patients had moderate UC. The reason for the higher rate of moderate and severe UC cases in the present study compared to the previous reports may be that some patients with mild UC who did not undergo a colonoscopy were included in the non-UC group in our study.

Patients with IBD associated with PSC are at a significantly increased risk of developing CRC, and the risk of CRC increases every year after a diagnosis of IBD [13]. A colonoscopy screening follow-up is therefore necessary. In our cohort (*n* = 77), two patients (5.7%) developed CRC. Twenty-one of the 35 patients diagnosed with UC were asymptomatic, and in 19 of these 21 patients, UC had been detected by a colonoscopy screening. These 19 patients comprised 56% of the patients who underwent a colonoscopy screening in the absence of symptoms of UC. These data indicate that PSC patients should undergo a colonoscopy for the detection of concurrent IBD regardless of whether they have symptoms of IBD. At our hospital, all PSC patients are advised to undergo a colonoscopy, but some refuse to do so or their poor general condition precludes a colonoscopy.

Fecal calprotectin or another marker might be helpful for selecting PSC patients who are most likely to benefit from colonoscopy screening. In the present study, we did not identify any clinical parameters that could be used to select PSC patients for colonoscopy screening; the patient's age at the diagnosis of PSC was the only parameter that differentiated the patients with and without UC. Of the patients diagnosed with UC, 24 (69%) were treated with 5-ASA, and 12 (34%) required treatment with PSL because their disease was moderate or severe. The prevalence of moderate or severe UC in our study is inconsistent with the previous finding that UC associated with PSC is mostly mild [10]. In PSC patients, even mild asymptomatic UC should be treated intensively to achieve early disease control and reduce the risk of CRC. Ideally, the use of PSL should be avoided in PSC patients because it may increase the risk of infections, such as infectious cholangitis. To minimize the risk of infection in this population, we have used CAP (cytapheresis), which has no known adverse effects on immune function, and we have observed the remission of UC after this therapy.

A recent study showed that mucosal addressin cell adhesion molecule 1 (MAdCAM-1) plays a role in the pathogenesis of PSC with UC [28]. Vedolizumab, a gut-selective integrin antagonist with no identified systemic immunosuppressive activity that prevents integrin binding to MAdCAM-1 on the gut mucosa, is one of the treatment options for IBD in patients with an underlying condition such as PSC. The prognosis of such patients is significantly affected by the success of infection control. Future studies should focus on developing treatments with no adverse effects on immune function.

### 4.2. The Prognosis of Patients with Both PSC and UC

PSC has a poor prognosis and is reported to have a 10-year transplantation-free survival rate of 54.9% [29]. In our entire patient cohort, the 10-year survival rate was higher at 69.1%. However, our PSC patients without UC had a lower 10-year survival rate (45.8%), whereas those with UC had a higher 10-year survival rate (92.7%). The non-UC group who did not undergo colonoscopies may have included patients with mild UC. We therefore recalculated the survival rate excluding the patients who did not undergo a colonoscopy. The results were almost identical, and there was no influence on the patients without colonoscopy. The patients with and without UC were diagnosed with PSC at different ages, and this is likely to have played a part in the major difference in survival between the two groups. To adjust for this difference in survival, a study that matches propensity scores should be performed in age-matched cohorts. However, since the age distribution of PSC is bimodal and UC is predominant in young people, it may be difficult to adjust the patient ages using age-matched cohorts. Compared to our present patients without UC, those with UC had nonsignificantly lower blood levels of hepatobiliary enzymes. These data suggest that PSC is more likely to be diagnosed at an earlier stage in patients with UC due to the presence of any symptom of UC or a diagnosis of UC. Hence, when examining patients with IBD, clinicians should consider the diagnostic possibility of PSC. Taking such an approach will lead to the early detection of PSC, which, together with the future development of effective treatments, will improve the prognosis of PSC.

This study has several limitations. The two groups of patients were not matched for age or sex, and this was a single-center, retrospective study. We also did not assess the diagnostic value of the fecal occult blood or calprotectin tests. Future studies should examine larger numbers of patients or be population-based, or propensity score matching should be used, as should various fecal tests for detecting UC.

Our findings contribute to the current research on IBD associated with PSC in several ways. This study is the first to report an increase in moderate cases of UC associated with PSC, and it is also the first to investigate the reasons for performing a colonoscopy in PSC patients and to show the importance of colonoscopy screening for detecting UC in this population.

## 5. Conclusions

In this retrospective analysis, the clinical and colonoscopic features of UC associated with PSC in the studied cohort are more moderate or severe UC than previous cases. Therefore, the effectiveness of treatments other than 5-ASA should also be evaluated. The coexistence of UC might affect the prognosis of PSC. There is also asymptomatic UC in patients with PSC. In this regard, screening for colonoscopy in PSC patients is essential. When a diagnosis of PSC is made, immediate colonoscopy is a priority with UC complications in mind. Future studies should address how to select PSC patients for colonoscopy screening.

## Figures and Tables

**Table 1:** Typical characteristics of ulcerative colitis and bowel inflammation associated with PSC [9–11].

	Ulcerative colitis (typical features)	Bowel inflammation associated with PSC
Site of inflammation	Inflammation spreads continuously from the rectum	Inflammation predominantly affects the right colon
Backwash ileitis	No	Yes
Rectal sparing	No	Yes
Distribution of inflammation	Diffuse	Patchy
Severity of inflammation:		
Mild	67%	76%
Moderate	29%	24%
Severe	4%	0%
Symptoms	Mucous/bloody stools, discomfort, etc.	None or mild symptoms

**Table 2:** Clinical characteristics of the PSC patients with concurrent UC (*n* = 35).

Gender, male : female	17 : 18
Age at diagnosis of PSC, yrs	36 ± 17
Age at diagnosis of colitis, yrs	36 ± 17
Initial diagnosis:	
PSC	10
Colitis	10
Both	15
Symptoms, yes : no	8 : 27
Site of inflammation:	
Total colitis (right-sided)	35 (30)
Left-sided colitis	0
Proctitis	0
Distribution:	
Patchy	30
Diffuse	5
Back wash ileitis:	
Yes	13
No	16
Unknown	6
Rectal sparing, yes : no	18 : 17
Colitic cancer	2
Liver biopsy Ludwig's stage:	
Stage I	2
Stage II	17
Stage III	3
Stage IV	1
Unknown	12
Lichtiger Clinical Activity Index (CAI)	4 (2-8)
Endoscopic UC severity score	
Mayo endoscopic score	1 (1-3)
Ulcerative Colitis Endoscopic Index of Severity (UCEIS)	2 (1-5)
Treatment^a^:	
Ursodeoxycholic acid	31
5-Aminosalicylic acid	24
Prednisolone	12
Granulocyte and monocyte adsorptive apheresis	5
Azathioprine	3

Data are the mean ± SD, median (min-max), or the number of patients. ^a^Some patients had more than one treatment.

**Table 3:** Clinical characteristics of the total PSC patient series.

	UC group *n* = 35	Non-UC group *n* = 42	*p* value
Gender, male : female	17 : 18	18 : 24	ns
Age at diagnosis of PSC, yrs	36 ± 17	55 ± 19	<0.0001
Colonoscopy, yes : no	35 : 0	20 : 22	<0.0001
Site of inflammation:			
Intrahepatic bile duct	8	7	ns
Extrahepatic bile duct	2	4	ns
Both	24	31	ns
Child-Pugh classification (A/B/C)	24/10/1	25/15/2	ns
Liver biopsy Ludwig's stage:			
Stage I	2	0	ns
Stage II	17	21	ns
Stage III	3	5	ns
Stage IV	1	1	ns
Unknown	12	15	ns
Treatment:			
Ursodeoxycholic acid	31	36	ns
At PSC diagnosis:			
PT, %	88.7 ± 10.3	93.5 ± 11.6	ns
CRP, mg/dl	1.55 ± 2.48	1.69 ± 2.45	ns
Alb, g/dl	3.73 ± 0.45	3.73 ± 0.55	ns
Total Bil, mg/dl	0.77 ± 0.40	1.69 ± 2.06	ns
AST, U/l	39.6 ± 20.7	69.5 ± 62.1	ns
ALT, U/l	47.0 ± 25.8	75.8 ± 83.0	ns
ALP, U/l	935 ± 884	1060 ± 834	ns
Cr, mg/dl	0.69 ± 0.19	0.80 ± 0.33	ns
Na, mEq/l	140 ± 2	13 ± 3	ns
PSC follow-up period (days)	3254 (229-11825)	1857 (173-9223)	0.0133

Data are the mean ± SD, median (min-max), or the number of patients. Alb: albumin; ALP: alkaline phosphatase; ALT: alanine aminotransferase; AST: aspartate aminotransferase; Bil: bilirubin; Cr: creatinine; CRP: C-reactive protein; Na: sodium; ns: not significant; PT: prothrombin time.

**Table 4:** Relationship between the reason for colonoscopy and UC (*n* = 55).

		UC		
		Yes	No	
Symptoms (including fecal occult blood positive)	Yes	16	5	21
	No	19	15	34

		35	20	55

## Data Availability

The datasets generated and analyzed during the current study are not publicly available due individual privacy could be compromised but are available from the corresponding author on reasonable request.

## Supplementary Materials

Supplementary Materials Supplementary Figure S1: survival rate (patients undergoing colonoscopy): PSC and PSC complicated by UC.
