# Supplementary figures and images for: Clinical Characterization of Ulcerative Colitis in Patients with Primary Sclerosing Cholangitis
Source: Gastroenterol Res Pract. 2020 Nov 7;2020:7969628. doi: 10.1155/2020/7969628 (PMC7669346; doi:10.1155/2020/7969628)

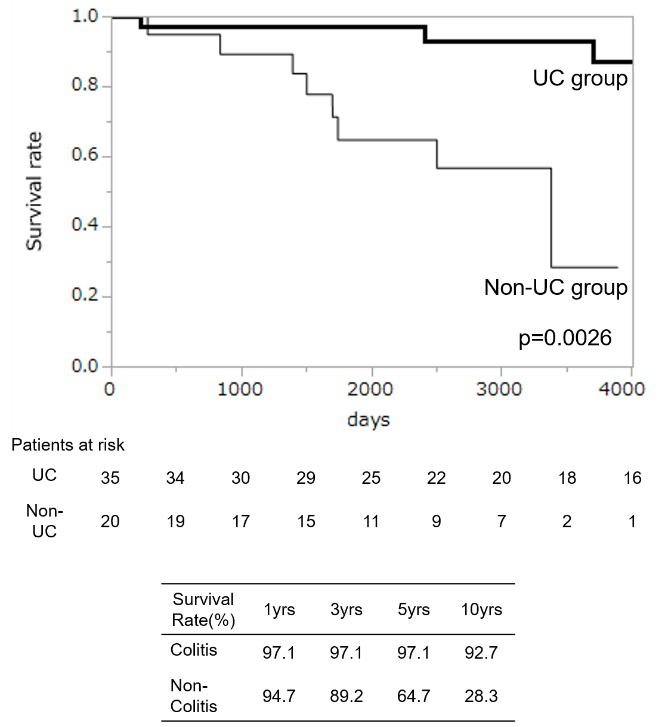


**Suppl. Fig. S1.** Survival rate (patients undergoing colonoscopy): PSC and PSC complicated by UC.

Supplement: Supplementary Materials — Supplementary Figure S1: survival rate (patients undergoing colonoscopy): PSC and PSC complicated by UC. [file 7969628.f1.docx]
